# Harnessing the untapped genetic diversity of local landraces: omics technologies as a gateway to horticultural breeding

**DOI:** 10.3389/fpls.2026.1920481

**Published:** 2026-09-10

**Authors:** Photini V. Mylona, Lorenzo Barchi, Luciana Gaccione, Annalisa Cocozza, Pasquale Tripodi, Ilias Avdikos

**Affiliations:** 1 Institute of Plant Breeding & Genetic Resources, ELGO-DEMETER, Thermi, Greece; 2 DISAFA, Plant Genetics, University of Turin, Turin, TO, Italy; 3 CREA Research Centre for Vegetable and Ornamental Crops, Pontecagnano Faiano, SA, Italy; 4 Laboratory of Vegetable Crop Science, Department of Agriculture, International Hellenic University, Sindos, Thessaloniki, Greece

**Keywords:** decision-support systems, genotyping, germplasm characterization, pangenome, phenomics, predictive breeding modules, trait-associated variants, variome

## Abstract

Horticultural crops, particularly Solanaceae and Cucurbitaceae, represent a major component of global vegetable production and are increasingly exposed to climate variability and environmental constraints. Landraces and crop wild relatives constitute essential reservoirs of adaptive genetic diversity; however, their effective utilization in breeding programs remains limited by fragmented characterization, incomplete passport information, and reliance on labor-intensive morphological descriptors. These limitations hinder the systematic exploitation of conserved germplasm and restrict its integration into modern predictive breeding frameworks. Recent advances in high-throughput phenotyping, genomics, and multi-omics technologies have created new opportunities to bridge the gap between genotype and phenotype. Next-generation phenomics enables non-destructive, high-resolution quantification of plant physiological and structural traits across environments, while genomic approaches, including whole-genome resequencing, support comprehensive assessment of genetic diversity. Pangenome frameworks further extend this resolution by capturing core and variable genomic fractions, collectively defining the species variome and enabling improved identification of structural and allelic variants associated with adaptive traits. The integration of phenomic and genomic datasets through multi-omics approaches enhances the functional interpretation of trait-associated variation and strengthens the predictive capacity of breeding strategies. In this context, the genome as a functional passport constitutes a unified reference layer linking germplasm identity with genomic, phenotypic, and functional trait information, thereby enabling more systematic germplasm characterization, reduced redundancy, and improved identification of elite parental material. This mini-review highlights how the integration of phenomics, pangenomics, and multi-omics enables the transition from descriptive germplasm cataloguing toward more systematic, data-driven, and predictive breeding systems for the development of climate-resilient horticultural crops.

## 1 Introduction

Over the last decade, the unprecedented pace of technological advancement has created new opportunities for agriculture and plant breeding to address the challenges posed by climate change and safeguard food security for a growing global population. Simultaneously, increasing emphasis has been placed on improving both the nutritional quality and sustainability of food production systems, particularly in horticultural crops, which constitute essential components of the human diet (Kathi et al., 2024; Zhao et al., 2024). Fresh fruits and vegetables play a fundamental role in human health, with members of the Solanaceae and Cucurbitaceae families, both predominantly annual crop groups, accounting for more than one-third of global fresh vegetable production, underscoring their pivotal contribution to modern agricultural systems. Among cultivated plant families, Solanaceae is widely recognized as one of the most economically important crop families worldwide, ranking only behind the dominant Poaceae and Fabaceae, while Cucurbitaceae represents one of the major vegetable crop families, encompassing globally important crops such as cucumber, melon, watermelon, pumpkin, and squash (Chomicki et al., 2020; Ma et al., 2022).

In this context, the identification and sustainable utilization of resilient genetic resources have emerged as strategic priorities, positioning traditional crop landraces at the forefront of research efforts aimed at enhancing agricultural sustainability, nutritional security, and climate resilience. Shaped over centuries by the combined effects of abiotic and biotic selection pressures, together with farmer-driven selection and local agricultural practices, landraces represent dynamic reservoirs of adaptive genetic diversity (Casañas Artigas et al., 2026). Although a substantial proportion of landrace diversity is currently conserved in *ex situ* genebank collections worldwide, many traditional landrace populations continue to be maintained within on-farm systems where they remain exposed to changing environmental conditions and farmer selection. These *in situ* populations retain their evolutionary capacity, generating novel genetic combinations through ongoing recombination and selection, thereby complementing the more static genetic resources preserved in genebanks (Mercer and Perales, 2010). The resulting diversity is continuously subjected to environmental and biotic challenges that shape agricultural productivity, crop performance, and product quality. Despite their considerable adaptive potential, however, landraces remain underutilized in modern breeding programs, largely due to the fragmented, labor-intensive, and resource-demanding nature of their characterization.

A major constraint to the effective utilization of landraces is the heterogeneous nature of the information available for conserved germplasm. Passport information, commonly standardized through the Multi-Crop Passport Descriptors (MCPD) framework (Alercia et al., 2015), remains incomplete, inconsistently documented, or restricted to basic taxonomic and geographic descriptors, despite substantial efforts toward data harmonization and improved genebank documentation (Hanson et al., 2025; Van Hintum et al., 2025). Furthermore, inaccuracies in taxonomic assignment, species misclassification, synonymy, and the presence of duplicated accessions across collections can further complicate germplasm management and utilization, potentially obscuring the true extent and distribution of genetic diversity preserved within genebanks (Tripodi et al., 2021; Barchi et al., 2023). Likewise, germplasm characterization has traditionally relied on standardized descriptor lists and labor-intensive field observations, which have provided the indispensable foundation for germplasm conservation, documentation, and utilization within genebank collections worldwide (Alercia et al., 2015; Weise et al., 2020; Engels and Ebert, 2021). However, phenotypic evaluations conducted across different locations, years, and experimental protocols often lack full comparability, while genotype-by-environment (G×E) interactions can substantially influence trait expression across environments/seasons. Furthermore, morphology-based assessments are inherently affected by phenotypic plasticity, whereby identical genotypes may express different phenotypes under contrasting environmental conditions, potentially obscuring underlying genetic variation and limiting the transferability of phenotypic observations across studies and environments (Schneider, 2022; Napier et al., 2023). Consequently, substantial reservoirs of adaptive genetic diversity remain insufficiently characterized, limiting their effective utilization in breeding programs and highlighting the need for more integrative, scalable, and biologically informed approaches to germplasm characterization.

The aim of this mini-review is to highlight the major bottlenecks and emerging technological advances influencing the effective utilization of the largely underexplored genetic diversity preserved within local landraces and germplasm accessions conserved worldwide, both in genebanks and in on-farm systems where continuous adaptation is shaped by natural and farmer-mediated selection under increasingly challenging climatic conditions. We examine how integrated, multidimensional characterization approaches encompassing advanced phenomics, transcriptomics, metabolomics, genomics, and pangenomics can bridge the gap between genotype and phenotype by systematically connecting phenotypic performance with the underlying genomic diversity conserved within germplasm collections. By enabling the functional interpretation of underutilized genetic resources, these approaches offer new opportunities to unlock the adaptive and breeding potential of landraces, thereby supporting the development of more resilient, productive, and sustainable horticultural cropping systems. Within this context, the emerging concept of the “functional passport” proposes genomic information as a stable and integrative reference layer for linking phenotypic, molecular, and evolutionary information across diverse datasets and environments. By transforming fragmented biological data into biologically meaningful and breeding-relevant knowledge, this framework can facilitate the identification, prioritization, and utilization of adaptive diversity preserved within landrace germplasm collections.

## 2 Integrating phenomics and pangenomics for functional germplasm characterization

### 2.1 Bridging morphology to next-generation phenomics

Currently, over 60% of major crop landrace groups are represented in *ex situ* repositories (Ramirez-Villegas et al., 2022), making their conservation essential for safeguarding genetic potential for future breeding programs. However, available documentation remains largely restricted to MCPD descriptors and/or basic phenotypic characterizations based on standardized international crop descriptor lists (Phillips et al., 2026), which are primarily designed to assess *ex situ* plant genetic resources or to establish distinctness, uniformity, and stability criteria.

Although standardized descriptor systems constitute the backbone of germplasm management and characterization, their reliance on discrete and often categorical traits inherently limits their capacity to capture the multidimensional nature of plant phenotypes and their dynamic responses to environmental conditions (Rosero et al., 2019; Weckwerth et al., 2020; Mondal et al., 2023; Zavafer et al., 2023). This limitation creates a significant bottleneck for large-scale plant phenotyping, as manual scoring remains highly time-consuming during both field and greenhouse evaluations, as well as in subsequent data curation. Furthermore, such traditional methodologies are restricted in throughput and are inherently susceptible to observer bias and human error (Kumar et al., 2015; Yang et al., 2020), making the evaluation of large, diverse germplasm collections economically and logistically unfeasible. Moreover, traditional descriptor frameworks predominantly target static, heritable morphological traits evaluated at specific, isolated developmental stages. Consequently, they are insufficient to resolve complex G×E interactions or to capture the dynamic, time-series physiological processes essential for understanding the adaptive mechanisms of local landraces under fluctuating environmental stresses (Araus and Cairns, 2014; Lasky et al., 2023; Zavafer et al., 2023; Rizwan et al., 2025). As climate change exacerbates the frequency and severity of abiotic and biotic challenges, these constraints severely limit the generation of high-resolution phenotypic datasets required to support informed selection strategies and to accelerate the development of resilient and environmentally adapted cultivars. To address these limitations, next-generation phenomics has emerged as a critical methodological advancement for capturing the complexity of plant performance beyond static, descriptor-based evaluations (**Table 1**). The past two decades have witnessed substantial advances in sensing technologies and the integration of engineering, plant science, and artificial intelligence, driving the emergence of modern crop phenotyping through high-throughput phenotyping (HTP) systems (Yang et al., 2020; Ahmad et al., 2025). Providing a scalable approach to overcome previous limitations, these integrated platforms bridge automated data acquisition with mechanistic trait interpretation, enabling the non-destructive extraction of complex physiological and structural plant traits (Yang et al., 2020), and facilitating continuous monitoring of dynamic responses to environmental stimuli. In this context, next-generation phenomics does not replace classical descriptor systems but rather extends them, providing a dynamic framework for linking plant performance with environmental variation and supporting a more refined characterization of GxE interactions.

**Table 1:** Schematic comparison of traditional phenotyping and next-generation phenomics for germplasm characterization.

Feature	Traditional phenotyping	Next-generation phenomics
Objective	Germplasm characterization using standardized descriptors	Quantitative, high-resolution characterization of dynamic plant traits for precision breeding
Traits	Morphological and agronomic descriptors assessed at discrete developmental stages	Morphological, physiological, biochemical, and stress-response traits monitored continuously
Data acquisition	Manual observations and measurements	Automated sensor platforms (RGB, multispectral, hyperspectral, thermal, LiDAR, chlorophyll fluorescence, GC-MS, etc.)
Temporal resolution	Single or few assessment time points	Continuous, longitudinal, and time-series monitoring
Environmental assessment	Limited evaluation of phenotypic plasticity and G × E interactions	Dynamic assessment under controlled and field conditions enabling detailed G × E analysis
Throughput	Low	High to ultra-high
Precision	Observer-dependent and susceptible to human bias	Objective, standardized, and highly reproducible digital measurements
Advantages	Simple and inexpensive. Internationally standardized. Suitable for routine germplasm characterization. Easy data interpretation.	Automated and non-destructive High throughput and high resolution Captures dynamic physiological responses and complex traits Suitable for precision breeding
Limitations	Labor-intensive and time-consuming Subjective assessments Low throughput Limited physiological information Mainly static trait evaluation	High infrastructure and instrumentation costs Bioinformatic expertise required Large and complex datasets Challenges in data standardization and integration
Applications	Germplasm characterization, DUS testing, variety description, assessment of morphological diversity	Large-scale germplasm screening, precision phenotyping, genomic prediction, crop physiology, early stress detection, candidate gene discovery, and predictive breeding

At their core, HTP technologies leverage light–plant interactions, where physiological and structural changes in plant tissues modify reflectance signatures across the electromagnetic spectrum, primarily within the visible (VIS) and infrared (IR) regions (Folta and Carvalho, 2015). The VIS domain (~400–700 nm) corresponds to wavelengths perceptible to the human eye, where leaf pigments actively absorb light, enabling optical data to reconstruct plant architecture and monitor growth dynamics and overall physiological status (Araus et al., 2022). In contrast, the IR domain (>700 nm) captures signals associated with water absorption, structural scattering, and thermal emission (Gerhards et al., 2019). Depending on spectral resolution and band configuration, imaging systems can be broadly classified into RGB, multispectral, and hyperspectral platforms. RGB systems acquire three broad visible bands and are primarily used for morphological and canopy-level assessments from emergence to harvest. Multispectral imaging captures multiple discrete visible and near-infrared (NIR) bands, while hyperspectral imaging provides the highest spectral resolution by recording hundreds to thousands of narrow and contiguous bands, generating a detailed spectral signature of plant status (Tripodi et al., 2022). Each platform offers distinct capabilities for high-resolution phenotyping: RGB systems are particularly effective for morphological trait quantification, whereas multispectral and hyperspectral approaches enable more detailed analyses of pigment composition, water status, and vegetation indices (Wu et al., 2023; Fan et al., 2026). Notably, hyperspectral approaches are increasingly being applied in Solanaceae and Cucurbitaceae systems, where genome-enabled trait dissection is supported by advanced spectral–genomic integration frameworks developed in recent pangenomic studies (Tripodi et al., 2022; Barchi et al., 2023).

Beyond reflectance-based imaging, thermal and chlorophyll fluorescence sensors provide real-time measurements of physiological processes and transient stress responses by capturing energy fluxes between plants and their environment (Pineda et al., 2020; Zavafer et al., 2020; Yang et al., 2025). The integration of spectral imaging with thermal and fluorescence sensing allows modern phenotyping platforms to resolve complex traits such as photosynthetic efficiency, transpiration regulation, and canopy temperature dynamics, enabling the detection of stress-induced physiological alterations well before the onset of visible symptoms (Jangra et al., 2021). Operationally, these technologies can be implemented in plant-to-sensor systems, where plants are transported via automated conveyor-based platforms to stationary imaging units, or in sensor-to-plant configurations, where mobile gantries move sensors across fixed plants (Cocozza et al., 2025; Du et al., 2025a). Both approaches enable high-throughput evaluation of large populations under controlled experimental conditions, including abiotic stress treatments such as drought, salinity, and nutrient variation. Notably, sensor-to-plant systems offer additional advantages for *in situ* phenotyping under field conditions, enabling direct assessment of crop performance in realistic environmental contexts. Complementary metabolomic and proteomic platforms, including GC-MS, LC-MS, and NMR-based approaches, provide detailed insights into metabolic pathways and biochemical networks underlying phenotypic variation (Yan et al., 2022). Although these approaches are often destructive and labor-intensive, they complement high-throughput phenotyping datasets by providing molecular-level validation and functional context, thereby contributing to a more comprehensive characterization of the plant phenome.

Despite these technological advances, a major challenge remains the extraction of actionable knowledge from large-scale, multi-source phenotypic datasets and their effective integration with genomic information for trait dissection in breeding contexts (Wang et al., 2024). To address this limitation, machine learning algorithms and computer vision approaches are increasingly employed to automate feature extraction from raw sensor data (Kamencay et al., 2024). These computational pipelines enhance the interpretation of complex multi-omics datasets and support the development of predictive models for precision breeding applications (Yan et al., 2022). Recent advances in artificial intelligence have further expanded the capabilities of high-throughput phenotyping beyond conventional machine learning approaches. Deep learning architectures, including convolutional neural networks (CNNs), recurrent neural networks (RNNs), vision transformers (ViTs), and more recently, foundation models, have substantially improved automated plant detection, organ segmentation, disease diagnosis, biomass estimation, and early stress identification directly from raw sensor data while minimizing the need for manual feature engineering (Yang et al., 2020; Murphy et al., 2024; Ubbens et al., 2025). By exploiting self-supervised learning and transfer learning across large multimodal datasets, these AI-assisted approaches enable robust phenotypic inference across species, environments, and imaging platforms, while facilitating the integration of phenotypic, environmental, genomic, transcriptomic, metabolomic, and proteomic information into predictive breeding models (Wang et al., 2026; Sangjan et al., 2025). Ultimately, these advances support digital phenotyping, genotype-to-phenotype prediction, and data-driven breeding decisions, thereby accelerating the identification and utilization of adaptive diversity conserved within landraces and germplasm collections (Xu et al., 2025).

**Figure 1:**
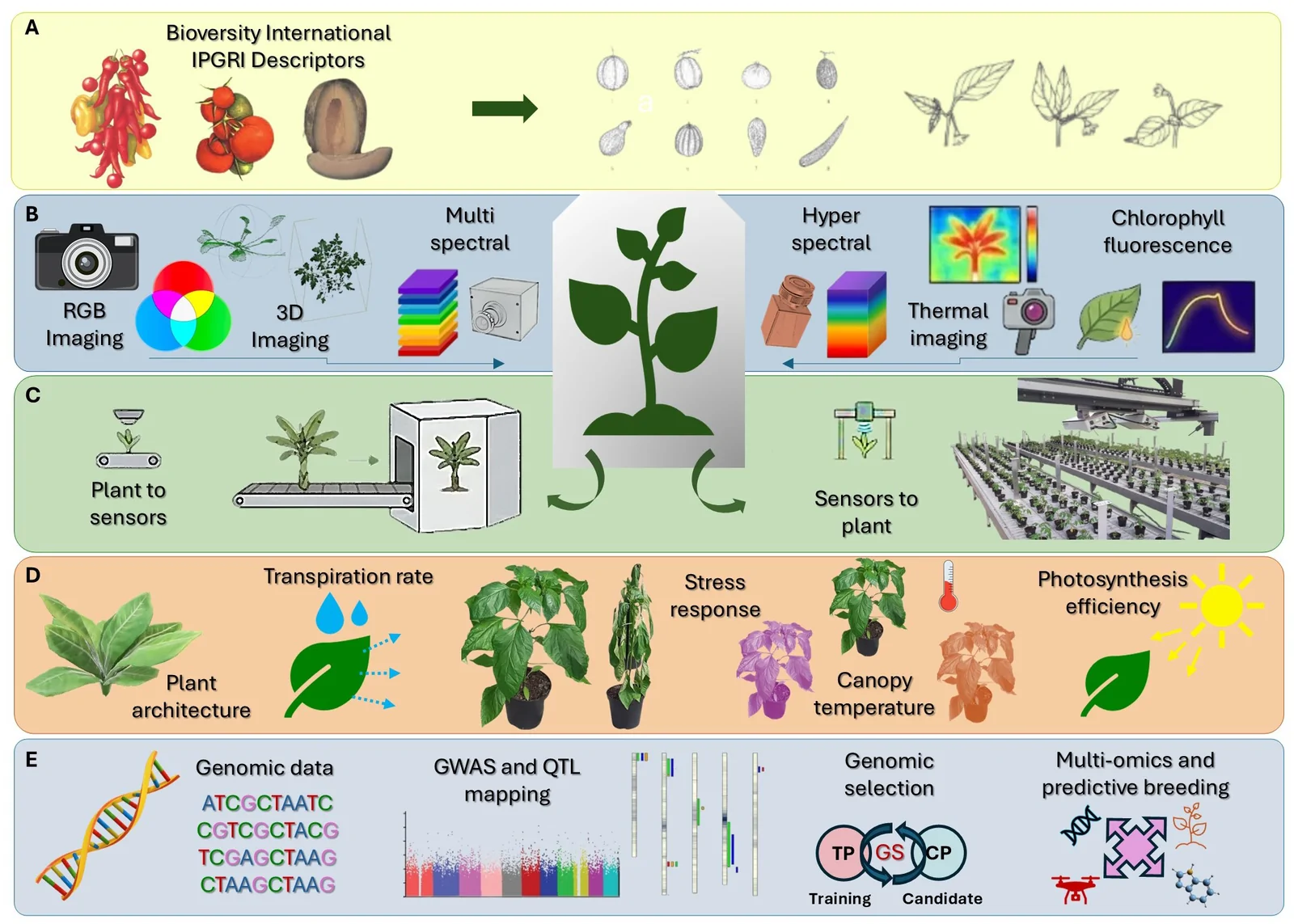
Evolution of plant phenotyping from classical descriptors to high-throughput systems and multi-omics integration for precision breeding. **(A)** Classical descriptors capture heritable morphological traits but poorly resolve environmental interactions. **(B)** High-throughput phenotyping integrates multisensor platforms (RGB, multispectral, hyperspectral, thermal, chlorophyll fluorescence) for early stress detection. **(C)** Plant-to-sensor and sensor-to-plant systems enable automated data acquisition. **(D)** Dynamic extraction of architectural and physiological traits enables real-time crop monitoring. **(E)** Integration with genomics and multi-omics supports candidate gene discovery and genomic selection.

Collectively, these advances establish high-throughput phenotyping as a scalable framework for capturing plant functional complexity in a quantitative and dynamic manner. The progression from manual descriptor-based evaluation to integrated imaging and sensor-driven platforms is summarized in **Figure 1**, highlighting the transition toward increasingly high-resolution and automated phenotyping systems. Yet, translating this phenotypic resolution into biological and breeding insight ultimately depends on integration with the underlying genomic architecture, which forms the basis for interpreting the genetic determinants of trait diversity explored in the following section.

### 2.2 Deep mining the pangenomic blueprint

The increasing availability of crop pangenomes is shifting horticultural genomics from marker-centered diversity assessment toward the functional interpretation of adaptive genetic variation. Reduced-representation genotyping approaches, including genotyping-by-sequencing (GBS), SPET, and SNP arrays, have significantly advanced the characterization of germplasm collections by interrogating thousands of loci at relatively low cost (Barchi et al., 2019, Barchi et al., 2023; Tripodi et al., 2021; Blanca et al., 2022; Pons et al., 2022; Lin et al., 2025). These approaches have supported studies on population structure, genetic relationships, introgression, and marker–trait associations but remain limited in their ability to capture the full spectrum of genomic diversity, particularly in landraces and crop wild relatives. Whole-genome resequencing provides substantially higher resolution, enabling the detection of both common and rare variants and supporting analyses of demographic history, selection, and genome-wide adaptation (Tripodi et al., 2022). However, reliance on a single linear reference genome can still obscure non-reference sequences, structural variants, copy-number variations, and presence/absence variants that often underlie adaptive phenotypes (He et al., 2025; Secomandi et al., 2025). Pangenomics addresses this limitation by integrating multiple high-quality assemblies into a unified representation of species diversity. In this framework, genomic content is partitioned into core regions shared across accessions and variable regions containing accession-, population-, or species-specific sequences. Together, these components define the species variome, encompassing the full spectrum of genetic diversity across individuals and populations, including SNPs, structural variants, copy-number variations, haplotypes, and presence/absence variation, spanning both cultivated and wild gene pools.

This is particularly relevant in horticultural crops, where landraces and wild relatives retain adaptive alleles frequently lost during domestication and modern breeding. Structural variants (SVs), including insertions, deletions, inversions, translocations, copy-number variations, and presence/absence variants, can influence gene dosage, regulatory architecture, chromatin context, and stress-response pathways, thereby contributing to phenotypic diversity not captured by traditional SNP-based marker technologies. Recent pangenomic resources in Solanaceae and Cucurbitaceae illustrate this transition, where graph-based and super-pangenome approaches have enabled structural-variant-aware association studies, improved genomic prediction, and identification of loci linked to fruit quality, salinity tolerance, pathogen resistance, and agronomic performance (Vaughn et al., 2022; Li et al., 2023; Cheng et al., 2025; Gaccione et al., 2025; Shi et al., 2026; Sun et al., 2026; Zhao et al., 2026). Similar advances have also been reported in pepper and eggplant, where graph-based pangenomes have facilitated the identification and functional characterization of structural variants associated with domestication, fruit quality, and adaptive responses to biotic and abiotic stresses, further demonstrating the broad applicability of pangenomic resources across horticultural crops (Barchi et al., 2023; Liu et al., 2023; Gaccione et al., 2025).

A central challenge moving forward is the transition from descriptive catalogues of pangenomic variation toward functional prioritization of variants relevant to adaptation and breeding. This requires integrated pipelines combining long-read assemblies, graph-based pangenome construction, and comprehensive genotyping of SNPs, SVs, CNVs, PAVs, and haplotypes across germplasm collections. Functional interpretation is further strengthened through the integration of transcriptomic, metabolomic, regulatory, and epigenomic information, enabling the identification of biologically meaningful variant–trait relationships. Recent multi-omics studies have demonstrated how integrating genomic, transcriptomic, and metabolomic datasets can reveal defense-associated pathways, regulatory networks, and metabolite accumulation underlying disease resistance and adaptive responses, thereby providing a functional basis for prioritizing breeding-relevant alleles (Iqbal et al., 2025). When integrated within coherent analytical frameworks and combined with pangenomic information, these complementary omics layers provide a foundation for improved interpretation of molecular variation and more informed breeding decisions (Varshney et al., 2021; Mascher et al., 2024). Graph-based approaches are particularly powerful in this context, as they enable sequence reads from diverse accessions to be aligned to a multi-genome reference rather than a single linear genome, thereby reducing reference bias and improving detection of non-reference alleles (Du et al., 2025b; Secomandi et al., 2025). This enables structural variants and haplotypes to be treated as explicit genomic features in downstream association and genomic prediction analyses. Comparative pangenomics further enhances the interpretation of under-characterized germplasm by leveraging conserved synteny and orthology across related assemblies. Through this approach, structural or variable genomic regions identified in poorly documented accessions can be linked to conserved gene networks involved in abiotic stress responses, developmental regulation, pathogen perception, and quality-related metabolic pathways. Such comparative frameworks have proven effective in tomato, eggplant, pepper, cucumber, and watermelon, where they have facilitated the discovery of adaptive loci associated with salinity tolerance, disease resistance, and fruit quality traits (Li et al., 2023; Liu et al., 2023; Gaccione et al., 2025; Shi et al., 2026; Sun et al., 2026).

**Figure 2:**
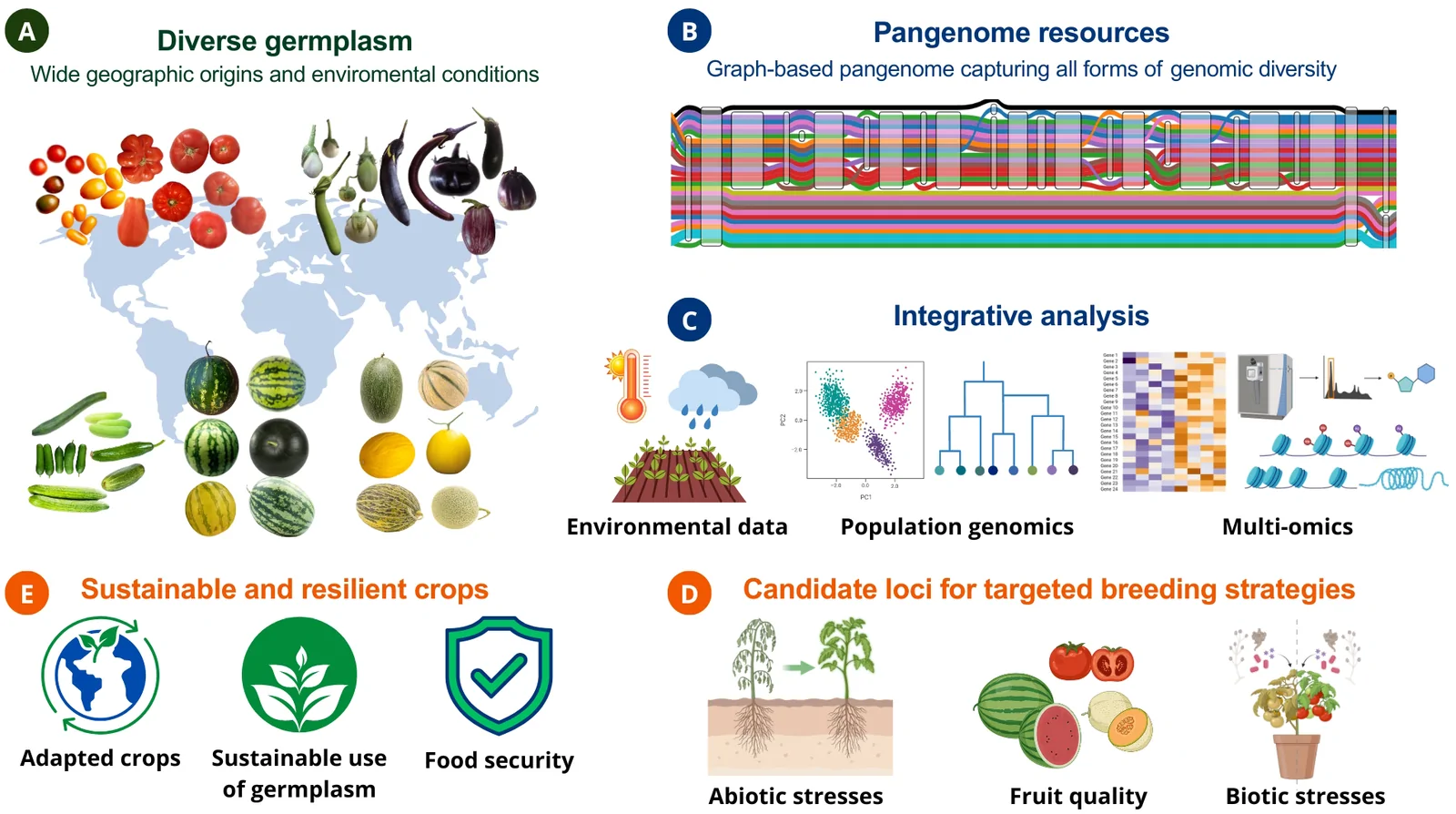
Pangenomics-enabled framework for germplasm exploitation in crop improvement. **(A)** Diverse germplasm from contrasting geographic and environmental origins. **(B)** Graph-based pangenomes capture genomic diversity beyond a single reference genome and define the species variome. **(C)** Integration with phenomics, environmental data, population genomics, and multi-omics enables identification of trait-associated loci. **(D)** Predictive breeding modules support germplasm utilization and parental selection. **(E)** Integrated data enable climate-resilient breeding strategies.

Population genomics adds a temporal and evolutionary dimension to this framework by integrating pangenomic variation with allele frequencies, haplotype structure, environmental metadata, and demographic history. This enables the identification of selective sweeps, local adaptation signals, and domestication-associated bottlenecks, thereby allowing the prioritization of variants with likely adaptive relevance (Schreiber et al., 2024). In several horticultural species, structural variants have been shown to coincide with regions under selection for drought, salinity, heat, and pathogen resistance, underscoring their role as reservoirs of adaptive diversity (Zhou et al., 2022; Gaccione et al., 2025; Shi et al., 2026; Sun et al., 2026). When integrated with high-throughput phenotyping and multi-omics datasets, pangenomic frameworks provide a direct bridge between observable stress-response traits and their underlying structural and regulatory determinants. Thus, the genome can increasingly function as a predictive biological passport for each accession, summarizing stress-associated haplotypes, structural alleles, candidate genes, and inferred breeding value. In this context, pangenome-informed decision-support systems, integrated with phenomic and multi-omics resources, offer a pathway toward more efficient and systematic exploitation of landrace and wild germplasm for climate-resilient horticultural breeding (**Figure 2**).

### 2.3 Bridging functional diversity to breeding applications

**Figure 3:**
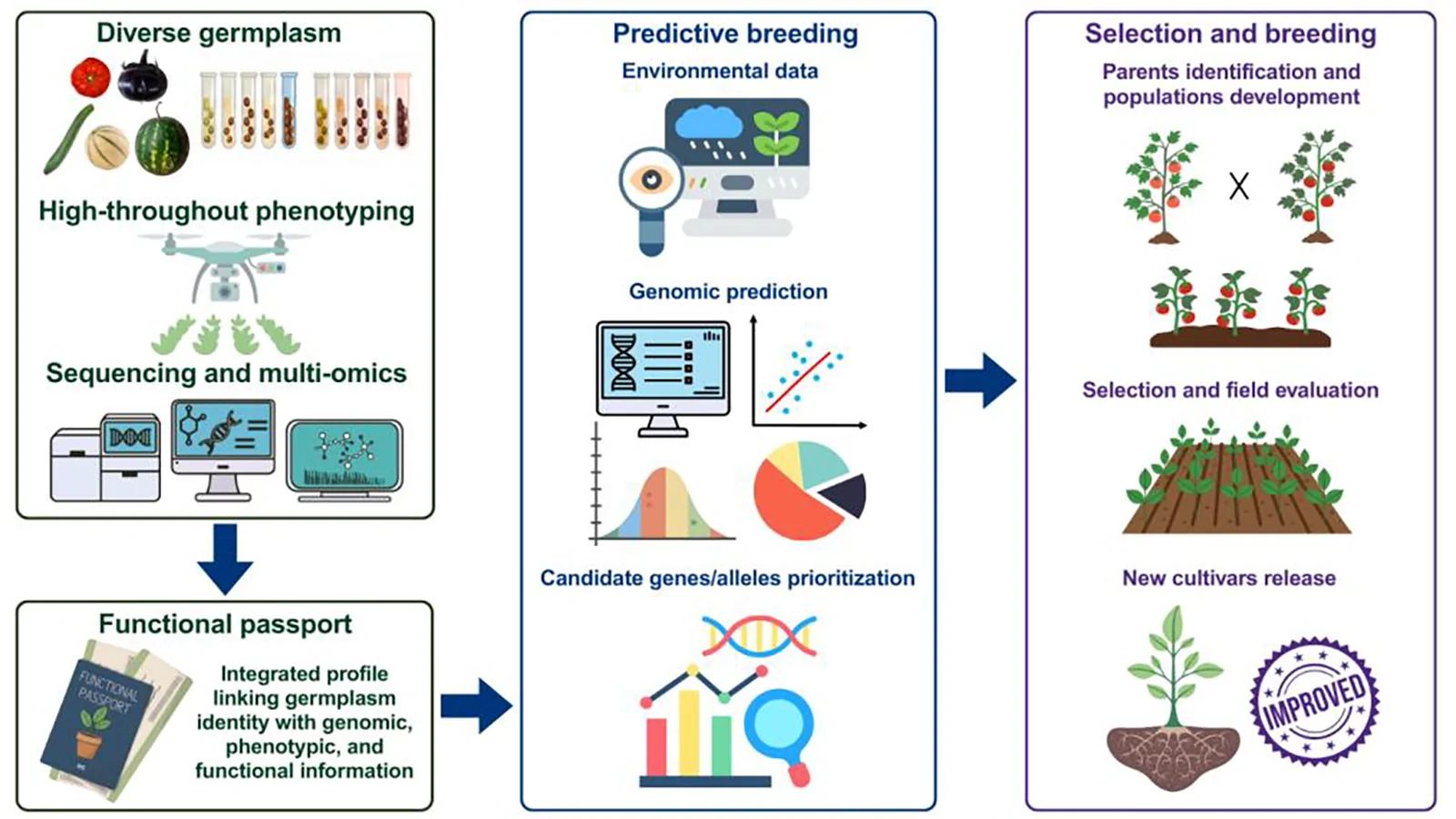
Schematic workflow from functional germplasm characterization to predictive breeding and cultivar development.

The integration of high-throughput omics technologies represents a decisive inflection point in horticultural breeding, transforming landraces from passive repositories of genetic variation into actionable biological resources. Despite major advances in sequencing and phenotyping technologies, traditional breeding remains constrained by a narrow genetic base and a modernization bottleneck that limits the efficient identification and deployment of adaptive traits into improved cultivars (Araus and Cairns, 2014). Over the past decade, extensive multi-omics datasets, including genome assemblies, population resequencing, transcriptome atlases, and metabolomic profiles, have been generated across major horticultural crops, particularly tomato, cucumber, and watermelon. However, these resources often remain fragmented and poorly interconnected, limiting their full translational potential. Recent studies demonstrate that integrating comparative genomics with transcriptomic and metabolomic analyses can identify candidate genes, regulatory pathways, and metabolic responses associated with disease resistance and environmental adaptation, thereby facilitating the functional prioritization of breeding-relevant loci (Iqbal et al., 2025). When integrated within coherent analytical frameworks and combined with pangenomic information, they provide a foundation for improved interpretation of molecular variation and more informed breeding decisions (Jamil et al., 2020; Varshney et al., 2021; Mascher et al., 2024). The overall workflow linking germplasm characterization with predictive breeding is summarized in **Figure 3**, illustrating how phenotypic, genomic, and multi-omics information can be integrated to support genomic prediction, candidate gene prioritization, parental selection, and cultivar development.

Within this context, pangenomics enhances the functional interpretation of genetic diversity by resolving gene clusters organized in syntenic blocks and linking structural variation to biochemical pathways and complex phenotypes. By capturing the full spectrum of allelic and structural diversity, pangenome-informed frameworks enable more accurate association between molecular variation and trait expression. Consequently, they provide a more complete basis for inferring genotype-phenotype relationships and improving parental selection strategies compared to single-reference approaches (Tettelin et al., 2005; Golicz et al., 2020; Li et al., 2023). A key challenge in predictive breeding, however, lies in the context dependence of trait expression under real-world field conditions. While multi-omics models can be highly informative under controlled conditions, GxE interactions and management variability may reduce the transferability of predictions across locations and seasons (De Los Campos et al., 2009; Cooper et al., 2016; Crossa et al., 2017). Addressing this limitation requires iterative model refinement through multi-environment validation and the incorporation of predictive frameworks capable of integrating spatial and temporal variability. In this setting, genomic information provides a stable reference layer that enables consistent integration of phenotypic and molecular datasets across environments. Rather than replacing environmental information, the genome functions as a structural anchor for organizing multi-omics data and supporting the development of predictive models of plant performance (Crossa et al., 2017). Building on this framework, breeding modules emerge as decision-support systems that prioritize elite parental materials based on integrated molecular and phenotypic evidence. These systems enable the identification of candidate genes and functional alleles associated with target traits, including both SNP- and structural-variant-based genomic features, and support the prediction of crosses with improved probability of favorable combining ability within genomic selection frameworks that increasingly incorporate pangenomic and multi-omics information (Meuwissen et al., 2001; Jannink et al., 2010; Poland et al., 2012; Hickey et al., 2017).

Accordingly, elite parental materials serve as entry points for multi-omics integration, where genotypic identity operates as a “genomic passport” linking phenotypic, transcriptomic, metabolomic, and pangenomic information within interoperable analytical environments. Within this framework, interoperable breeding modules integrate genomic selection outputs with multi-omics and pangenome-derived information to enable systematic cross-ranking of parental materials based on predicted breeding value and allelic complementarity. This structure is further strengthened by the concept of the “functional passport,” which provides a unified reference layer linking germplasm identity with genomic, phenotypic, and functional trait information. The inclusion of regulatory information, including noncoding RNAs, can further improve the functional characterization of breeding-relevant alleles. For example, Liu et al. (2026) demonstrated that the rice blast resistance gene *Pish* is associated with reprogramming of long noncoding RNAs during interaction with *Magnaporthe oryzae*, linking resistance to regulatory networks involved in stress responses and phytoalexin biosynthesis. This example highlights the value of integrating functional genomics with molecular regulatory mechanisms to improve the interpretation and prioritization of breeding-relevant variation. Despite their considerable potential, the implementation of integrated multi-omics and pangenomic breeding frameworks remains constrained by heterogeneous data formats, incomplete metadata, inconsistent ontologies, and differences in analytical pipelines, which limit data integration and interoperability across databases and breeding programs (Papoutsoglou et al., 2020, Papoutsoglou et al., 2023; Selby et al., 2025). Furthermore, pangenome construction and multi-omics analyses require substantial computational infrastructure, data storage, bioinformatic expertise, and long-term data curation, while sequencing and high-throughput phenotyping remain costly, particularly for smaller breeding programs (Hu et al., 2024; Du et al., 2025b). Equally important is the adoption of reproducible analytical workflows and standardized metadata to ensure that genomic and phenotypic datasets remain comparable, reusable, and interoperable across studies, environments, and breeding programs. Addressing these challenges will require the continued adoption of community standards, FAIR data principles, interoperable frameworks such as MIAPPE and BrAPI, and scalable computational infrastructures capable of supporting collaborative data sharing, analysis, and decision-making across breeding networks. Collectively, this architecture supports more informed decision-making in parental selection and enhances breeding efficiency by transforming dispersed biological data into actionable, prediction-oriented knowledge for climate-resilient crop improvement (Meuwissen et al., 2001; Jannink et al., 2010; Crossa et al., 2017; Hickey et al., 2017).

The integration of phenomics, pangenomics, and multi-omics datasets establishes a continuous framework linking genetic variation to phenotypic expression and breeding decisions. Phenomics contributes high-resolution functional characterization of plant performance, while pangenomics captures the full spectrum of structural and allelic diversity underlying adaptive traits. Their convergence enables a more complete and mechanistic interpretation of genotype–phenotype relationships beyond single-reference and static-trait paradigms (Tripodi, 2022; Wang et al., 2024). Organizing these interconnected data streams around breeding-relevant entities transforms dispersed biological information into structured decision-oriented knowledge. Within this framework, the genome as a ‘functional passport’ can serve as an accession-centered, rather than trait-specific, operational framework for linking germplasm identity, molecular variation, and trait performance across datasets and environments.This integrated approach facilitates more efficient prioritization of parental material, improves the resolution of trait-associated loci, and accelerates the utilization of underexploited genetic diversity in horticultural breeding systems (Hickey et al., 2017). Together, **Figures 1** and **2** define a phenome–genome continuum underpinning this framework. Recent pangenomic resources in Solanaceae and Cucurbitaceae illustrate this transition, where graph-based and super-pangenome approaches have enabled structural-variant-aware association studies, improved genomic prediction, and identification of loci linked to fruit quality, salinity tolerance, pathogen resistance, and agronomic performance (Vaughn et al., 2022; Li et al., 2023; Cheng et al., 2025; Gaccione et al., 2025; Shi et al., 2026; Sun et al., 2026; Zhao et al., 2026).

## 3 Conclusions and future perspectives

This mini-review highlights how the integration of high-throughput phenomics, metabolomics, genomics, and pangenomics provides a coherent framework for improving the characterization and utilization of landrace diversity in horticultural crops. Within this perspective, the genome as a “functional passport” emerges as a unifying concept for translating conserved but underutilized genetic resources into actionable knowledge for future climate-resilient breeding strategies. Beyond conceptual integration, these approaches also offer practical advantages for germplasm management by enabling more efficient characterization of collections, reducing redundancy, improving data coherence, and refining the resolution of underlying genetic structure within conserved diversity. Collectively, this framework enables a transition toward a more systematic, data-driven, and predictive paradigm for the exploitation of plant genetic resources in climate-resilient horticultural breeding. Over the next five to ten years, major research priorities will include the development of scalable graph-based pangenome references for population-level variant discovery, transparent and validated AI-assisted breeding tools, and FAIR, interoperable platforms linking germplasm with genomic, phenotypic, multi-omics, and environmental information. Equally important will be the establishment of integrated breeding platforms that connect genebanks with trait discovery, genomic prediction, and field validation, enabling more efficient translation of conserved genetic diversity into climate-resilient cultivars. Achieving this vision will require shared community standards, robust computational infrastructure, equitable access to advanced technologies, and close collaboration among researchers, breeders, and genebank curators.
